# Ultra-efficient Amplification of Abnormal Prion Protein by Modified Protein Misfolding Cyclic Amplification with Electric Current

**DOI:** 10.1007/s12035-017-0431-8

**Published:** 2017-02-13

**Authors:** Jeong-Ho Park, Yeong-Gon Choi, Seok-Joo Park, Hong-Seok Choi, Eun-Kyoung Choi, Yong-Sun Kim

**Affiliations:** 1 Department of Microbiology, College of Medicine, Hallym University, Chuncheon, Gangwon-do 24252 Republic of Korea; 2 Laboratory of Transmissible Spongiform Encephalopathies, Ilsong Institute of Life Science, Hallym University, 15 Gwanpyeong-ro, 170beon-gil, Dongan-gu, Anyang, Gyeonggi-do 14066 Republic of Korea; 3 Department of Biomedical Gerontology, Graduate School of Hallym University, Chuncheon, Republic of Korea; 4 Laboratory of Cellular Aging and Neurodegeneration, Ilsong Institute of Life Science, Hallym University, Anyang, Republic of Korea; 5 Korea CJD Diagnostic Center, Hallym University, Anyang, Republic of Korea

**Keywords:** Prion disease, CJD, Diagnosis, ePMCA, Sonication

## Abstract

Prion diseases are clinically diagnosed and confirmed upon post-mortem histopathological examination of brain tissue. The only reliable molecular marker for prion diseases is abnormal prion protein (PrPSc), a pathologically conformed prion protein that primarily accumulates in the central nervous system and to a lesser extent in lymphoreticular tissues. However, the use of PrPSc as a marker for preclinical diagnoses is limited because the concentration of PrPSc in easily accessible body fluids is extremely low. Hence, one of the most promising approaches would be the development of an efficient in vitro amplification method for PrPSc. Indeed, protein misfolding cyclic amplification (PMCA) has become an important diagnostic tool for prion diseases. Here, we first describe a new superior PMCA device that employs electricity (referred to as ePMCA) to amplify PrPSc. The ePMCA device markedly improved the detection limit for PrPSc by amplifying trace amounts of pathogenic prion protein by applying electricity to improve PMCA. To increase the cavitation of sonication, a glass sample tube was used, and the upper side of the horn was shaped such that it had a curved cross-section. The ePMCA device enabled PrPSc to be amplified even from a sample seeded with 10–28-fold diluted 263K scrapie-infected brain homogenates with recombinant hamster prion protein (rHaPrP). In addition, the efficiency of prion amplification was best when 50 mM HEPES and 1% Triton X-100 were used as a PMCA conversion buffer in the various conditions that we applied. These results indicate that ePMCA would be very valuable for the rapid and specific diagnosis of human prion diseases and, thus, may provide a practically improved method for antemortem diagnoses using the body fluids of patients and animals with prion disease.

## Introduction

Transmissible spongiform encephalopathies (TSEs), or prion diseases, make up a group of neurodegenerative disorders that include Creutzfeldt–Jakob disease (CJD) in humans, bovine spongiform encephalopathy (BSE) in cattle, and scrapie in sheep [1, 2]. The etiological agent of prion disease is an abnormal prion protein (PrP^Sc^), which is an abnormal isoform that is converted from the normal cellular protein (PrP^C^) by unknown post-translational modification processes. To date, human prion diseases are confirmed only by detecting PrP^Sc^ via post-mortem histopathological examination of brain tissues. The diagnosis of CJD is difficult because PrP^C^ is not sufficiently converted to PrP^Sc^ at the early stage of prion disease. Thus, various trials have been conducted to develop a diagnostic method with high sensitivity and accuracy for detecting trace amounts of PrP^Sc^ [3–5]. In recent studies, diagnostic methods that amplify pathogenic PrP^Sc^, such as protein misfolding cyclic amplification (PMCA) and quaking-induced conversion (QUIC), have been investigated [5–8]. Since PMCA was first reported in 2001, many studies based on PMCA have been conducted [3, 9–16]. Some researchers have detected PrP^Sc^ in body fluids such as the cerebrospinal fluid, blood, and urine, which can be more easily obtained than the brain tissue [9–14], and early diagnoses in animals using blood samples have been demonstrated [10, 11]. In 2011, a new technique referred to as PMCAb, wherein Teflon beads (McMaster-Carr, Los Angeles, CA) were added to the PMCA reaction to increase the efficiency of PrP^Sc^ amplification, was developed [15]. Many research teams were later able to detect pathogenic prion protein in specific prion infectious body fluids, such as the urine, blood, and saliva, utilizing PMCA devices [10–13, 16]. Therefore, we attempted to conduct experiments using a Misonix model S-4000 sonicator to amplify PrP^Sc^; however, more advanced methods for the early diagnosis of prion diseases need to be developed, as there is a lack of consistency among the results of experiments that have used PMCA devices. We sought to improve the amplification of pathogenic prion protein by using electricity (termed ePMCA). Here, we show that this technical improvement can be employed to overcome the limitations unique to conventional PMCA, i.e., that the amplification efficiency of PMCA strongly depends on the tube position, which results from the differential cavitation induced by the different distances of each tube from the horn center [15]. The improved ePMCA technique can also be used to simultaneously amplify PrP^Sc^ from very high dilutions of infected brain homogenate. The aim of this study was to determine the conditions that stimulate PrP^Sc^ amplification using the ePMCA technique and a conversion buffer. Supplementing the procedure with electricity resulted in remarkable improvements in the yield and reproduction rate of prion conversion and in the sensitivity of PrP^Sc^ detection. Here, we show that simple modification of the PMCA procedure can be implemented to overcome the drawbacks associated with PMCA and that ePMCA can be utilized for the early diagnosis of prion disease in animals and humans.

## Materials and Methods

### Animals and Scrapie Strains

We used 6-week-old inbred mice (C57BL/6J and ICR) and golden Syrian hamsters (SHa) from the SLC (Japan), which were divided into a group for infection with scrapie strains and a group of age-matched controls. The original stock of scrapie strains was provided by Dr. Alan Dickinson (Neuropathogenesis Unit, Edinburgh, Scotland). All passages were performed by intracerebral inoculation with 30 μl (mice) or 50 μl (SHa) of 1% (*w*/*v*) brain homogenates in 0.01 M phosphate-buffered saline (PBS, pH 7.4) obtained from the scrapie-infected brains at the terminal stage of the disease. We isolated the brains from the experimental animals when clinical symptoms were clearly evident, which was at 70 days post-inoculation (dpi) for the 263K scrapie-infected hamsters and at 158 dpi for the ME7 scrapie-infected mice. For Western blots, the brains from scrapie-infected animals and age-matched controls were frozen without perfusion and stored at −70 °C prior to use. Each experiment was repeated at least three times. All animal experiments were conducted in accordance with the guidelines set forth by the National Institutes of Health (NIH guide for the Care and Use of Laboratory Animals, USA) and by the Hallym Medical Center for Institutional Animal Care and Use Committee. The brains were placed in the chosen conversion buffer during each experiment and homogenized gently using a glass homogenizer (Corning, USA) to a concentration of 10% (*w*/*v*). Crude brain homogenates were centrifuged at 1500 rpm for 30 s at 4 °C. The supernatants were used as brain homogenates.

### Recombinant Prion Protein

Recombinant hamster PrP (rHaPrP), a plasmid containing DNA sequences coding for residues 23–231 of the hamster PrP sequence, was expressed, refolded into a soluble form, and purified as described previously [7]. We amplified DNA sequences coding for hamster PrP residues 23–231 by PCR, ligated them into the pET41 vector (EMD Biosciences, USA) as NdeI-HindIII inserts, and verified their sequences. After transforming the plasmids into *Escherichia coli* BL21 (DE3) cells (EMD Biosciences, USA), we expressed host-encoded cellular prion protein (PrP-sen) and lysed cell pellets with BugBuster and lysonase (EMD Biosciences, USA) in the presence of EDTA-free protease inhibitors (Roche, Germany), washed the recombinant prion protein (rPrP)sen inclusion bodies twice with 0.1 × BugBuster, and pelleted them by centrifugation. We purified the enriched rPrP according to a previously described method [8] with some minor modifications. We loaded a Ni-NTA Superflow resin (Qiagen, Germany) with denatured protein from the inclusion bodies and refolded the protein with a linear gradient over 6 h at a flow rate of 1.0 ml/min using an AKTA Purifier system (GE Healthcare, USA). We then eluted the rPrP proteins with 100 mM sodium phosphate (pH 5.8), 500 mM imidazole, and 10 mM Tris. We filtered and dialyzed them against 10 mM phosphate (pH 5.8) and then determined the concentration of rPrP. Aliquots of the proteins were stored at −70 °C after purification.

### The Combination of Surfactants and Buffer Solution

The buffer solution used for the PMCA method was termed as a conversion buffer, as previously described [17]. We performed the PMCA method using different buffer solutions, such as 2-(*N*-morpholino)ethanesulfonic acid (MES), piperazine-*N,N″*-bis(2-ethanesulfonic acid) (PIPES), *N*-2-acetamido-2-aminoethanesulfonic acid (ACES), 3-(*N*-morpholino)-2-hydroxypropane sulfonic acid (MOPSO), *N*-tris(hydroxymethyl)methyl-2-aminoethanesulfonic acid (TES), *N-*2-hydroxyethylpiperazine-*N*″-2-propane sulfonic acid (HEPPS), and HEPES, all containing sulfonic and carboxylic acid functional groups. The composition of the conversion buffer was as follows: 150 mM NaCl, 50 mM HEPES pH 7.0, 1% Triton X-100, and an EDTA-free protease inhibitor cocktail (Roche, Germany) per 50 ml of conversion buffer [18]. We performed the experiments using different concentrations of these HEPES buffer solutions ranging from 50 to 500 mM.

### PMCA Techniques: Conventional PMCA, Ilsong PMCA, and ePMCA

To avoid contamination, the preparation of noninfectious material was conducted inside a biological safety cabinet in a prion-free laboratory, and aerosol-resistant tips were used. The bench, pipettes, and other equipment were cleaned frequently with alcohol or bleach. The resulting 10% normal mouse brain homogenate in conversion buffer was used as the substrate in the Ilsong and Misonix S-4000 PMCA reactions. To prepare seeds, 10% scrapie brain homogenate in conversion buffer was serially diluted 10^3^- to 10^8^-fold, as indicated, in the conversion buffer, and 10-μl samples of the dilutions were used to seed 90 μl of normal mouse brain homogenate in the Ilsong and Misonix S-4000 PMCA devices. Samples in thin-wall 200-μl PCR tubes were placed in a rack fixed inside the Ilsong and Misonix S-4000 microplate horns. The sonicator was programmed to perform 96 repeated cycles of 1 min of sonication followed by 29 min of incubation, and the output was set to 50%. One round of the PMCA procedure consisted of a 48-h reaction comprising 96 cycles of sonication and incubation. The PMCA method consists of the repetition of 1 cycle of incubation and sonication. Electricity at 24 V DC/30 W was applied to the glass tube for 29 min, and then the tube was incubated at 37 °C in a water circulation system, and the ultra-wave sonication was adjusted during the last 1 min. We optimized the power of ultra-wave sonication by 50% to fit the experiment. At temperatures over 37 °C, the moisture was increased and evaporated into the lid of the tube, causing an imbalance in the inside of the tube and experimental samples. Thus, the inside of the tube, including the experiment samples, is not constant, and the amplification efficiency decreases as the content at the bottom of the tube upon applying the ultra-wave becomes thicker due to evaporation. Generally, 1 cycle consists of 30 min and 96 cycles, which is considered one round. Using 10 μg/ml of rHaPrP as a substrate, we performed PMCA (ePMCA, Ilsong PMCA, and Misonix S-4000 devices) with 100-fold diluted 263K scrapie-infected brain homogenates (10^−6^ to 10^−28^) in conversion buffer. The ePMCA reaction was performed in thin-wall 1-ml glass tubes. We made a slight modification to the PMCA experimental conditions described previously [17]. We chose a complex of piezoelectric elements, such as PbO, TiO_2_, ZrO_2_, Sb_2_O_3_, Nb_2_O_5_, and MnO_2_, among ceramic piezoelectric elements to use as an ultrasonic transducer. We designed a converter that emits 20 kHz and controls the output power from 0 to 2000 W if a 60-Hz electric signal is applied. We also fabricated a booster and a horn of titanium. To improve the existing PMCA, we designed an “Ilsong PMCA” device for maintaining the temperature of the cup horn via a water circulation system with a protection box. Additionally, we checked the temperature of the water tank in real time as we installed the temperature sensor in the cup-horn water tank.

### Detection of PrP^Sc^ by Western Blot

Infected brain tissues were treated with proteinase K (PK; 50~200 μg/ml final concentration; for 30 min at 45 °C in a shaker at 150 rpm). Additionally, we compared the PK resistance of brain PrP^Sc^ in the different conditions by changing the concentration of PK, the temperature regime of the proteinase K treatment, and the anti-prion antibodies (3F4 and 3F10) [19]. An insoluble fraction of brain tissue extract from a hamster or a mouse infected with the scrapie strain was heated at 100 °C for 10 min and electrophoresed (15% acrylamide gels) at 80 V. After electroblotting and applying a blocking buffer (5% skim milk in Tris-buffered saline (TBS); 1 h at room temperature), the nitrocellulose membranes were washed in TBS containing 0.05% Tween-20 (TBST) and incubated with anti-prion monoclonal antibodies (3F10 or 3F4, 1:3000 in TBST) and then with a horseradish peroxidase-conjugated secondary antibody in TBST containing 5% skim milk for 1 h at room temperature. At the end of each step, the membranes were washed four times for 10 min with TBST, and then the blots were visualized using a SuperSignal chemiluminescent substrate (Thermo Fisher, USA). The expression levels of each protein were quantified by densitometric analysis (Image Quant LAS 4000 mini, GE Healthcare, USA).

## Results

### Implementation of the Improved PMCA (Ilsong and ePMCA)

**Fig. 1:**
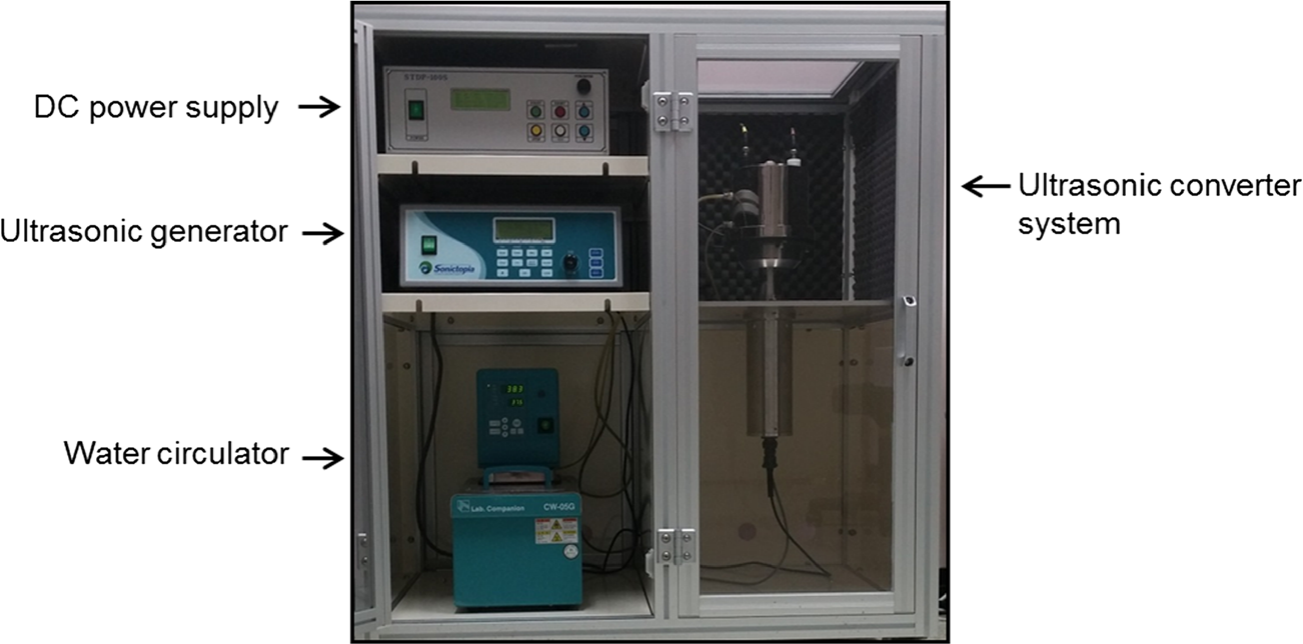
A photograph of the ePMCA device. We developed an improved PMCA device, termed ePMCA, that delivers an electric current. The device is composed of a DC power supply, an ultrasonic generator, a water circulator, an ultrasonic converter system, and a protection case

**Fig. 2:**
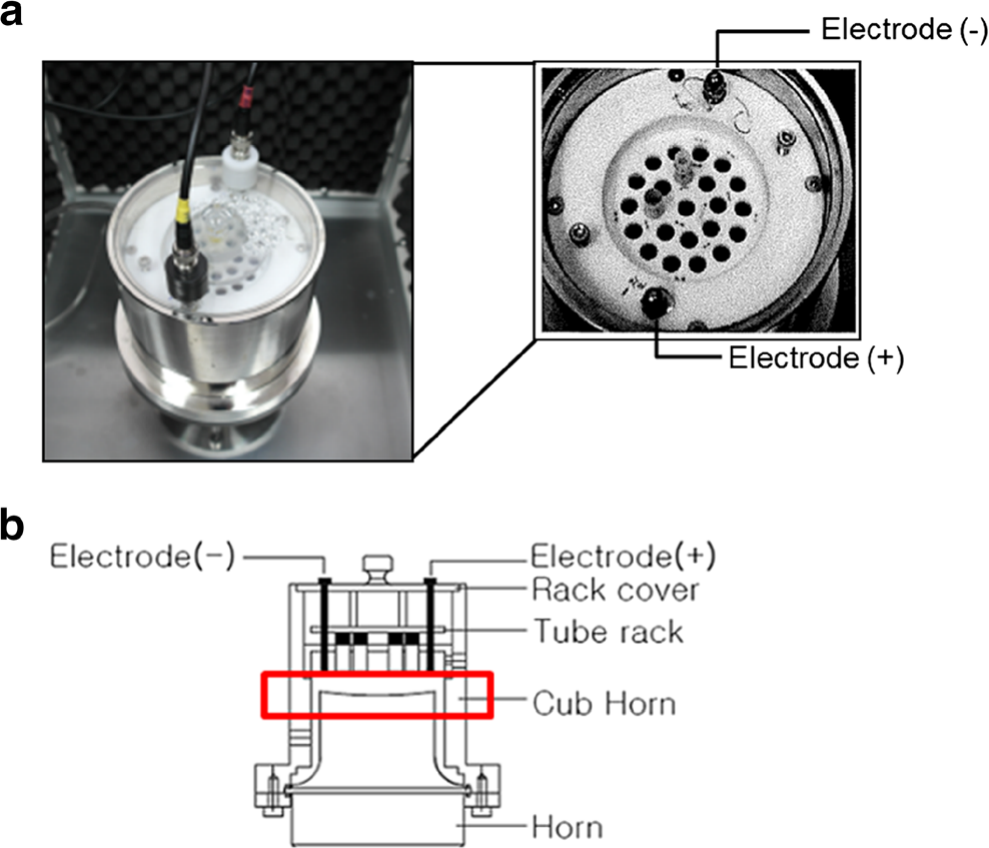
A diagram showing the installation of the cup horn, the tube plate, and the electric equipment in the ePMCA device. DC 24 V/30 W electric current (electrode +/−, **a**) is delivered during the period of incubation for the ePMCA reaction but not during the period of sonication. The cub horn in the ePMCA is hemispherically cross-sectioned (**b**)

To check the change of temperature during the PMCA reaction, we performed conventional PMCA (Misonix S-4000) in an air-cooling incubator at 37 °C for 10 to 15 consecutive days and monitored the water temperature in the cup horn. We found that the water temperature immediately increased to 50~60 °C after 10 cycles (data not shown), which may affect prion amplification in the later rounds of PMCA. Therefore, to maintain the water temperature during the conventional PMCA reaction, we installed a water-cooling system (a heating and water circulation system) that could control the water temperature in the cup horn by circulating the water through the horn (Ilsong PMCA). In addition, we applied electric current to the Ilsong PMCA device via water circulation system, which we termed ePMCA (Figs. 1 and 2). The horn in the ePMCA device was made of titanium, and a converter was also made of titanium to carry the strong output to the horn. The plastic tube that is generally used in conventional PMCA reactions was replaced with a glass tube in the ePMCA reaction to increase the transmissibility of cavitation. We sought to minimize the differential prion amplification in each glass tube in the ePMCA reaction by curving the top of the cup horn (Fig. 2) and thus finally developed an advanced ePMCA device to catalyze protein misfolding reactions (Fig. 1).

### Detection of PrP^Sc^ in the Scrapie-Infected Brains

**Fig. 3:**
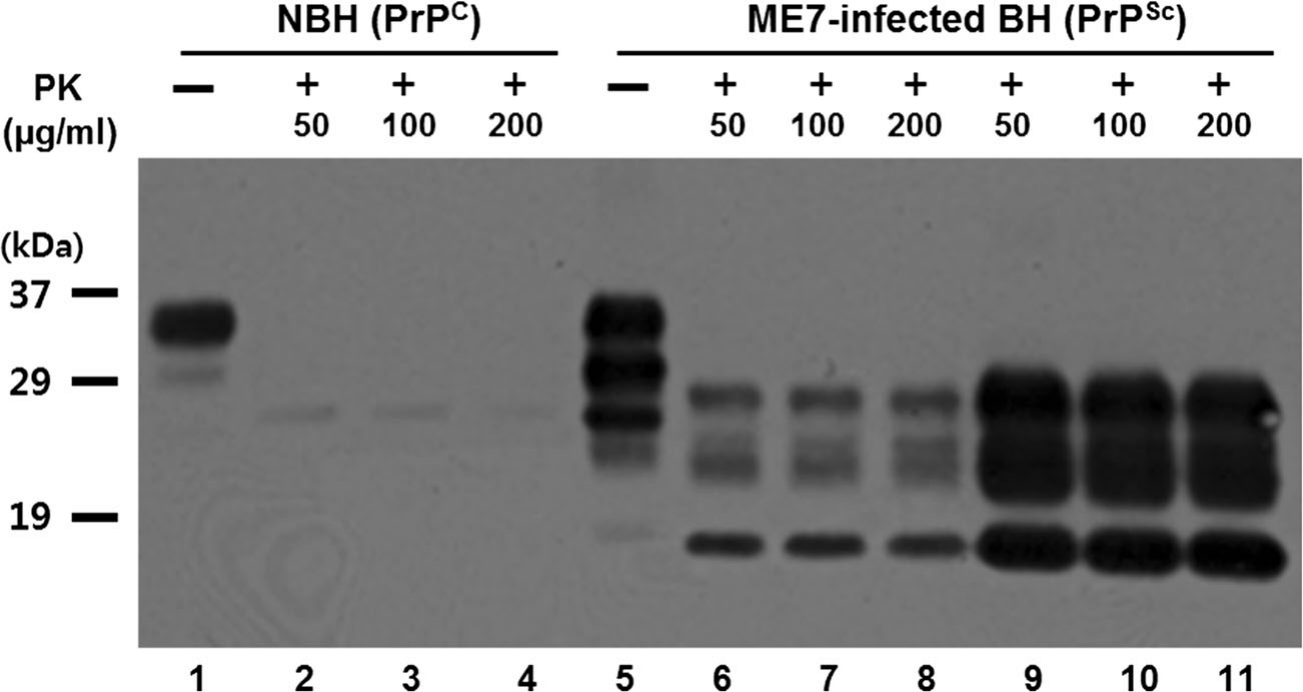
PK-resistant PrP^Sc^ was detected in the ME7-infected mouse brain. Western blotting was performed to test for the presence of PrP^Sc^ in the ME7-infected BH. *Lane 1* 0.05% NBH, *lanes 2–4* 10% NBH, *lane 5* 0.05% ME7 BH, *lanes 6–8* 0.1% ME7 BH, *lanes 9–11* 0.5% ME7 BH. *PK(+)*, PK treated; *PK(−)*, PK untreated. Molecular masses are indicated at the *left side* of the figure (kDa). Seventy micrograms/thirty microliters of total protein was loaded for each lane

Prior to starting the PMCA, we tested for the presence of PrP^Sc^ in ME7 and 263K scrapie-infected brain homogenates (BHs) by Western blot analysis. PrP^Sc^ accumulation was detected with the 3F10 antibody in both the ME7-infected and 263K-infected BHs (data not shown), whereas none was detected in normal brain homogenate (NBH) (Fig. 3). Two concentrations of brain tissues (0.1 or 0.5% BH) obtained from the ME7-infected mice were treated with PK at concentrations of 50, 100, or 200 μg/ml. We detected PK-truncated PrP^Sc^ in the infected brains, whereas PrP^C^ was completely degraded in the normal brains (Fig. 3). We observed a similar pattern of PrP^Sc^ bands in both brain preparations (0.1 or 0.5% BH) (Fig. 3).

### The Influence of the Buffer Solution and Surfactant on the Rate of PrP^Sc^ Amplification

**Fig. 4:**
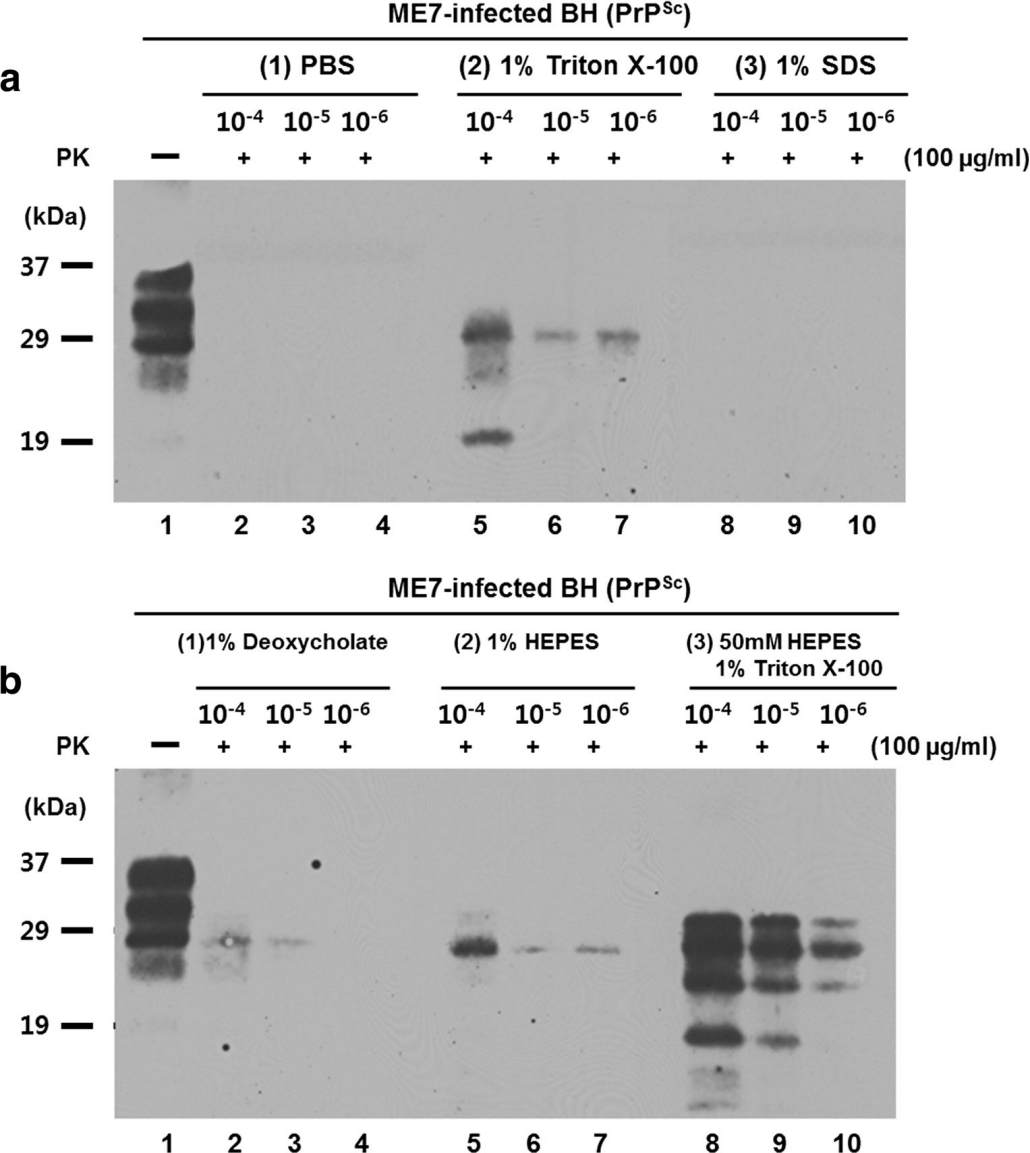
The effect of the buffer solution and the surfactant on the amplification of PrP^Sc^ derived from ME7-infected mouse brains. After ME7-infected mouse brains were individually homogenized in the buffers [**a** (*1*) PBS, (*2*) 1% Triton X-100 (PBS + 1% Triton X-100), and (*3*) 1% SDS (PBS + 1% SDS); **b** (*1*) 1% deoxycholate (PBS + 1% deoxycholate), (*2*) 1% HEPES (PBS + 1% HEPES), and (*3*) 50 mM HEPES and 1% Triton X-100], the homogenates were diluted by 10^−4^-fold to 10^−6^-fold with the same individual buffer. The diluted homogenates were used as prion seeds with the indicated buffer as the conversion buffer using mouse NBH as a substrate in the Ilsong PMCA device for one round, and then PrP^res^ was checked. *Lane 1*: 10^−3^-fold diluted ME7 BH was not subjected to PK digestion and PMCA. *Lane 2~10*: PK treated (100 μg/ml). Molecular masses are given on the *left* of each figure (kDa)

We investigated whether prion amplification can be influenced by the chemical compounds in the buffer solution in PMCA reactions by examining the differences in prion amplification based on the buffer solution. We performed one round of PMCA in various buffer compositions using the Ilsong PMCA device with ME7-infected BH as a seed and mouse NBH as a substrate (Fig. 4). As shown in Fig. 4a, when PBS containing NaCl, EDTA, and a complete protease inhibitor cocktail with 1% Triton X-100 (2), a nonionic surfactant, was used in the PMCA reaction, amplified PrP^Sc^ in the ME7-infected BH was observed. However, there was no amplification in the PBS-only buffer (1) or in the PBS buffer with 1% SDS (3) as one of the nonionic surfactants. Fig. 4b (1) shows a weaker band when PMCA was conducted upon adding 1% deoxycholic acid (1), a negative ion surfactant that has a steroid structure, than when Triton X-100 was used (Fig. 4a (2)). As shown in Fig. 4b (2), upon using 1% HEPES in PMCA, there was a stronger band compared to the use of deoxycholic acid and a weaker band compared to the use of Triton X-100. Fig. 4b (3) shows that PrP^Sc^ in the ME7-infected BH was more efficiently amplified by 50 mM HEPES with 1% Triton X-100 than with any other buffer composition. These results indicate that the effectiveness of prion amplification was improved when 50 mM HEPES with 1% Triton X-100 was used as the PMCA conversion buffer. We thus used the 50 mM HEPES with 1% Triton X-100 in the PMCA reactions described hereafter.

### Ultra-sensitive Detection of PrP^Sc^ by ePMCA

**Fig. 5:**
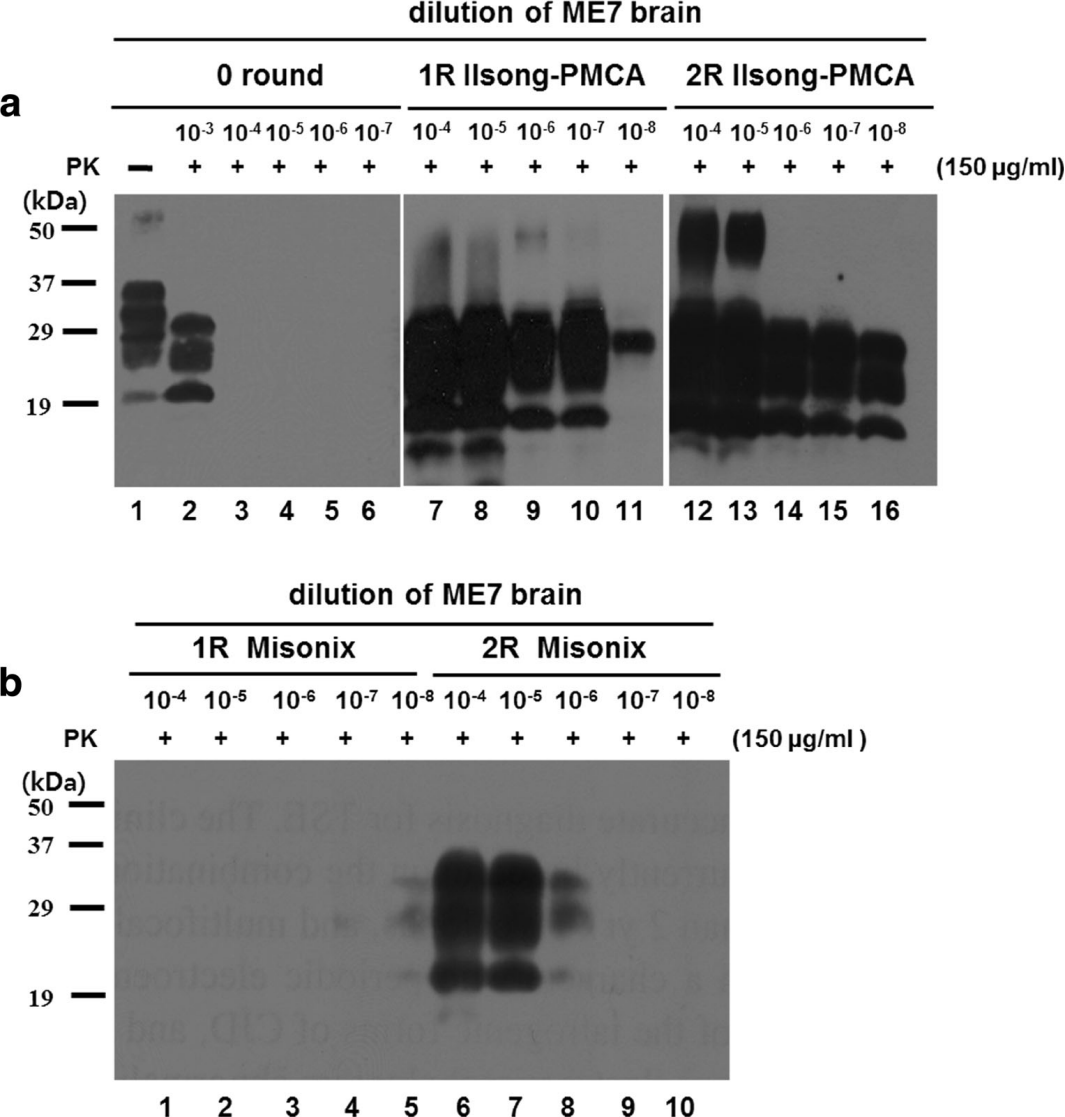
The Ilsong PMCA device improved the amplification of PrP^Sc^. Brain homogenates (10% wt/vol) prepared from ME7-infected mice in the terminal stage of scrapie were tenfold diluted with the 50 mM HEPES buffer from 1 × 10^−3^ to 1 × 10^−8^. The resulting NBH (10% wt/vol) extracted from healthy mice in HEPES buffer was used as the substrate in the PMCA reactions [Ilsong PMCA (**a**) and Misonix S-4000 PMCA (**b**)]. Samples (10^−4^ to 10^−8^ seeds) were subjected to one or two rounds of PMCA using the Ilsong and Misonix S-4000 devices. ME7 BH that was not subjected to PMCA was diluted by 10^−3^- to 10^−7^-fold with 50 mM HEPES buffer containing 1% Triton X-100 as shown in the left figure of the panel **a**. All samples were treated with PK (150 μg/ml). *Lane 1* of **a** indicates 0.5% mouse NBH (20 μg/30 μl). *PK(+)*, PK treated; *PK(−)*, PK-untreated. The samples were probed with the anti-PrP monoclonal antibody 3F10. Molecular mass markers are indicated in kilodalton on the *left*

Using BH of an ME7-infected mouse as a prion seed and mouse NBH as a substrate of prion conversion, we compared the efficiency of the infectious PrP amplification of the two devices, the conventional PMCA device and the new PMCA (Ilsong PMCA) device designed by us. When we used the conventional PMCA device, we detected resistant PrP to PK treatment in the second round (2R, 96 h) but not in the first round (1R, 48 h) (Fig. 5b). However, upon using the Ilsong PMCA device, we detected PK-resistant PrP (PrP^res^) in the first round of the reactions in all diluted ME7 BH (10^−4^- to 10^−8^-fold) (Fig. 5a). The PrP^res^ was detected in the 10^−3^-fold diluted BH of the ME7-infected mouse that was not subjected to PMCA (0 round) but not in the 10^−4^- to 10^−7^-fold diluted BHs (left of Fig. 5a). These observations indicated that the Ilsong PMCA device could induce better prion amplification than the conventional PMCA device.

**Fig. 6:**
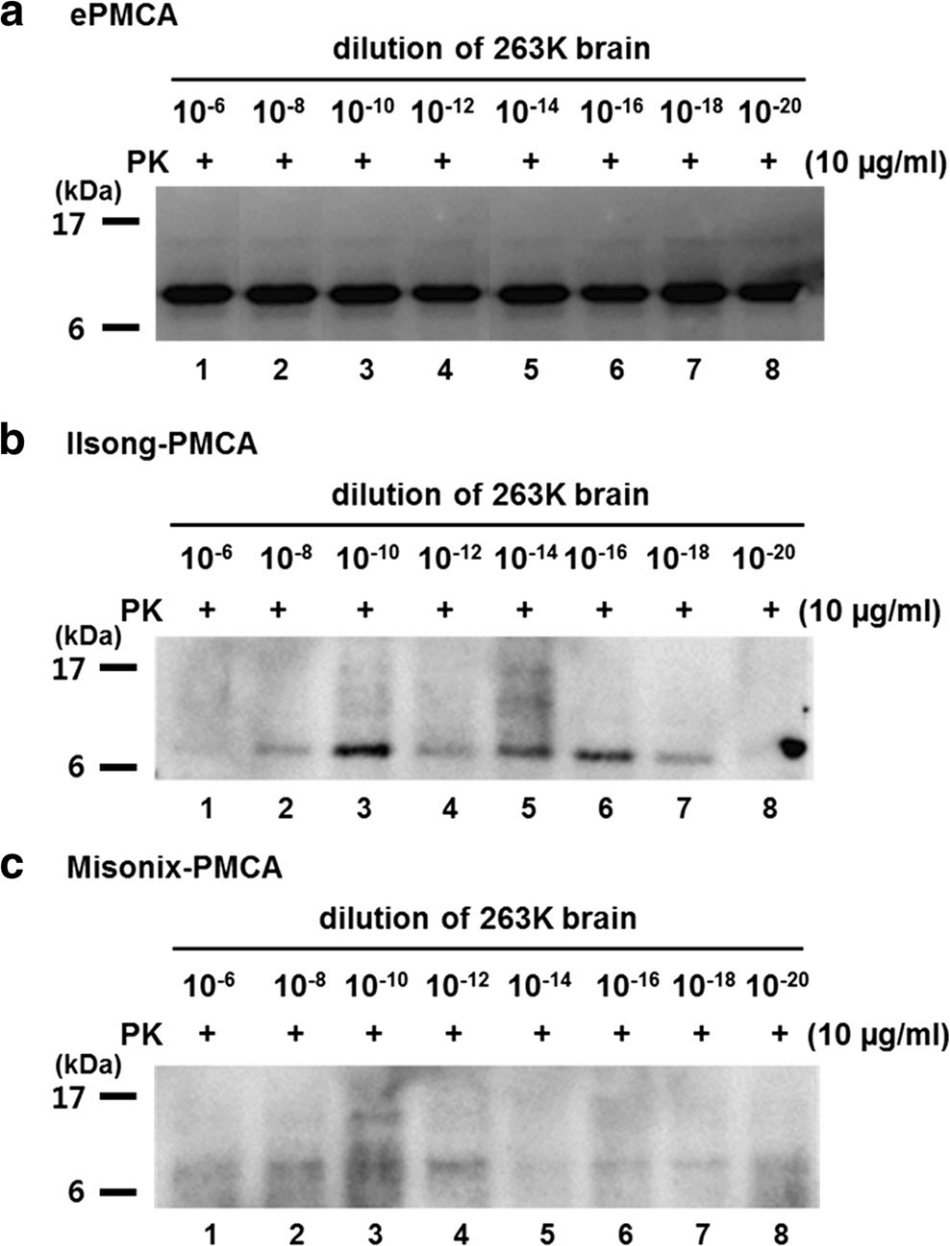
The ultra-efficiency of the ePMCA device to detect PrP^Sc^. 263K-infected hamster brain was homogenized and then 100-fold diluted from 10^−6^ to 10^−20^. PMCA was performed using the ePMCA (**a**), Ilsong PMCA (**b**) or Misonix S-4000 (**c**) devices. The diluted 263K BH was tested in 10 μg/ml of rHaPrP as a substrate. In *lanes 1~8* in each panel, 1.5 rounds of PMCA (ePMCA, Ilsong, and S-4000 devices) were performed in 10^−5^- to 10^−19^-fold diluted 263K BH (10 μl) with 10 μg/ml of rHaPrP (90 μl). All samples were digested with PK (10 μg/ml for 1 h at 37 °C) and analyzed using the 3F4 antibody. Molecular masses (kDa) are given on the *left*

Then, we compared the efficiency of PrP amplification among the three devices (Misonix S-4000, Ilsong PMCA, and ePMCA devices) using 263K-infected BH as a prion seed and rHaPrP as a substrate for 1.5 rounds. The three devices made it possible to amplify 10^−20^-fold diluted 263K-infected BH, and thus, one form of PrP^res^ between 6 and 17 kDa was detected, even though the intensities of the PrP^res^ were variable (Fig. 6). As shown in Fig. 6, compared to the Ilsong and Misonix S-4000 devices, the ePMCA device showed a stronger PrP^res^ amplification at the same concentration of the seed (10^−6^- to 10^−20^-fold diluted 263K-infected BH). These observations indicated that PrP^res^ formation occurred much more efficiently in the ePMCA device.

**Fig. 7:**
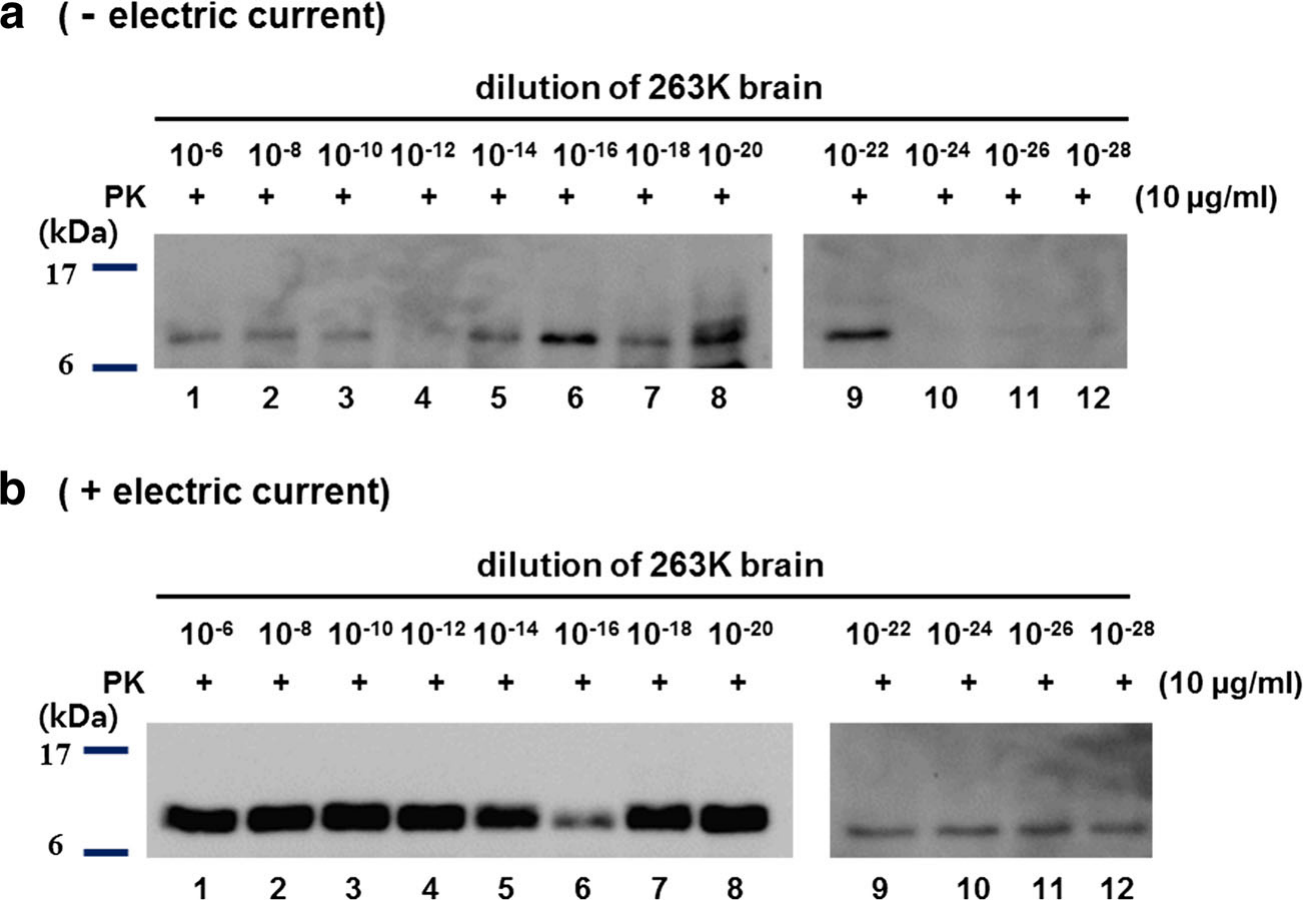
Electric current improved the rate of PrP^Sc^ amplification. 263K-infected hamster brain was homogenized and then 100-fold diluted by 10^−6^- to 10^−28^-fold. PMCA was conducted in the absence (**a**) or presence (**b**) of the electric current in the ePMCA devices. In lanes *1~12* in each panel, 1.5 rounds of PMCA were performed using the 10^−5^- to 10^−27^-fold diluted 263K BH (10 μl) with 10 μg/ml of rHaPrP (90 μl). All samples were digested with PK (10 μg/ml for 1 h at 37 °C) and analyzed using the 3F4 antibody. Molecular masses (kDa) are given on the *left*

To check the effect of the electric current on the PMCA reaction, we had PrP^Sc^ in the 263K-infected BH amplified for 1.5 rounds (72 h) using rHaPrP as a substrate in the presence or absence of electricity in the ePMCA device. After PMCA without applying the electric current (sonication for 1 min every 30 min, 144 cycles, no electric current), one form of PrP^res^ between 6 and 17 kDa was clearly detected to the 10^−22^-fold diluted 263K BH but very faintly detected in the 10^−24^- to 10^−28^-fold diluted BH (Fig. 7a). However, in the presence of the electric current, the form with the same size between 6 and 17 kDa was detected even in the 10^−28^-fold diluted 263K BH (Fig. 7b). These observations indicate that the electric current markedly strengthened the prion amplification in the ePMCA reaction.

## Discussion

In the present study, we demonstrate for the first time that the prion amplification via PMCA can be improved by applying an electric current to the PMCA device. The application of electricity increased the amplification rate of PK-resistant prion protein at a concentration of 10^−28^ within 1.5 rounds (Fig. 5b). The ePMCA device made it possible to detect the PK-resistant prion isoform in 10^−28^-fold diluted BH compared to detection in 10^−20^-fold diluted BH by the Ilsong PMCA device and detection in 10^−20^-fold diluted BH by the Misonix S-4000 PMCA device (Fig. 6). These results indicate that the ePMCA device allows for more efficient prion conversion and amplification of trace of pathogenic prion protein in the brain tissue used as a prion seed compared to conventional PMCA devices. As a result, because the best prion amplification was achieved by using ePMCA, ePMCA can be a useful device for detecting trace amounts of PrP^Sc^ in body fluids, such as the cerebrospinal fluid, blood, and urine.

Here, we demonstrated that the appropriate modification of the incubation process of in vitro prion amplification, i.e., the application of electricity, dramatically accelerates PrP^Sc^ amplification in the 263K strain. In addition, we also sought to test whether prion conversion can be chemically affected. The results showed that the conversion of PrP^C^ to PrP^Sc^ proceeded more easily upon adding buffer solutions with sulfonic acid and carboxylic acid functional groups at concentrations ranging from 50 to 500 mM compared to that obtained by using PBS. This observation may be due to the increased solubility of PrP^Sc^ upon use of a nonionic surfactant because PrP^Sc^ structurally has low solubility and a high rate of β-sheets. In this study, the efficiency of prion amplification increased the most when 50 mM HEPES buffer with 1% Triton X-100 was used as the ePMCA conversion buffer. Additionally, to solve the problem of the tube position-dependent variation in the prion amplification caused by each tube receiving slightly different sonication power, which frequently occurs in conventional PMCA devices, we modified the upper side of the horn to have a curved cross-section in the ePMCA device. Thus, the ePMCA device equipped with a curved horn can be used as a new diagnostic tool that allows for ultra-sensitive detection of PrP^Sc^ even in short rounds of ePMCA when HEPES buffer is used as a conversion buffer. ePMCA also makes it possible to hypersensitively detect PrP^Sc^ in less rounds (1.5 rounds, 72 h) than in conventional PMCA, which was reflected by the stronger intensity of the PrP^res^ band (Fig. 6). The results of our study show that under optimal PMCA conditions (sonication and modified incubation with an electric current), ePMCA is capable of detecting PrP^Sc^ in scrapie-infected BH diluted by 10^−28^-fold as the seed within a maximum of 1.5 reaction rounds. Using the conventional PMCA device, the temperature of the water in the cup horn increased to 50~60 °C immediately after sonication (data not shown), which may result in protein modification. To avoid protein modification at high temperatures, we developed two PMCA devices (Ilsong PMCA and ePMCA) that allow for the temperature of the water in the cup horn be kept constant by using a water circulation system. Furthermore, to increase cavitation, we used a glass sample tube, and the upper side of the cup horn was curved in the ePMCA device.

It remains unclear how electricity influences prion conversion, but we hypothesize that the process of incubation with electricity might help accelerate the rate of efficient conversion of PrP^C^ to PrP^res^, making prion conversion conditions more optimal than in the other PMCA reactions without electricity. In contrast, the presence of electricity may help rearrange the PrP^Sc^ propagation and, thus, may induce the breakdown of PrP^Sc^ polymer particles. Although the ePMCA device helps improve the sensitivity of prion detection in body fluids, such as human CSF or tears, the mechanism by which electricity is involved in prion propagation remains to be elucidated. In this study, we developed the world’s first early diagnostic method for prion diseases, the ePMCA device, which may be capable of detecting traces of pathogenic prion protein in prion-infected fluids. Therefore, we suggest that the development of a diagnostic method that can diagnose probable CJD patients or animals using body fluid samples is the most promising strategy for controlling the spread of the disease.

In conclusion, our findings suggest that ultra-sensitive detection of the PrP^Sc^ can be achieved using ePMCA. Even trace amounts of PrP^Sc^ can be detected using the new device, showing the superiority of the ePMCA device to detect PrP^Sc^ at higher sensitivities than any other PMCA device and further increasing the likelihood of the development of realistic diagnostics for CJD and BSE.
